# Exploring the Diverse Immune and Genetic Landscape of Psoriatic Arthritis

**DOI:** 10.3390/jcm10245926

**Published:** 2021-12-17

**Authors:** Bogdan Batko

**Affiliations:** Department of Rheumatology and Immunology, Faculty of Medicine and Health Sciences, Andrzej Frycz Modrzewski University, 30-705 Krakow, Poland; bpbatko@gmail.com

**Keywords:** psoriatic arthritis, inflammation, interleukin-17, interleukin-23, pathomechanism, modulation, immune

## Abstract

Psoriatic arthritis (PsA) is characterized by delays in diagnosis and modest effect of treatment in terms of joint response. An understanding of molecular pathomechanisms may aid in developing diagnostic and prognostic models. Genetic susceptibility (e.g., HLA class I genes, IL-23-related genes) can be responsible for the pattern of psoriatic manifestations and affinity for tissue involvement. Gene expression analysis indicates an inflammatory profile that is distinct for PsA, but disparate across tissues. This has clinical implications, as for example, dual blockade of IL-17A and IL-17F can lead to superior clinical effects if there is differential expression of IL-17 receptors in tissues. Structural and functional impairment of barrier tissue, including host-microbiome interactions, may be the source of immune activation. Interplay between different cell populations of innate and adaptive immunity is emerging, potentially providing a link between the transition of skin-to-joint disease. Th17 subsets, IL-17A, IL-17F and IL-23 are crucial in PsA pathogenesis, with both clinical and experimental evidence suggesting a differential molecular landscape in cutaneous and articular compartments.

## 1. Introduction: Burden of Psoriatic Arthritis and Importance of Early Diagnosis

Psoriatic arthritis (PsA) is a complex, multi-system disease that includes both articular and extra-articular features. Of the musculoskeletal manifestations, enthesitis, dactylitis, peripheral arthritis and axial joint involvement are common [1]. The burden of PsA is shaped by frequent hospitalizations and comorbidity, which contribute to a chronic and progressive natural history that often impairs daily life [2,3,4]. The economic burden of psoriatic arthritis is more substantial than that of psoriasis (PsO), with PsA associated with increased healthcare expenditures, necessity for more frequent sick leave and increase in physical disability [5]. 

The diagnosis of PsO usually precedes a diagnosis of PsA. Average age of onset is approximately 30 to 50 years [6]. Prevalence shows no apparent sex predilection and increases with the time since diagnosis, with some studies reporting rates of 5% at 20 years, while others have observed prevalence of over 20% at 30 years [2,6]. The pooled prevalence and incidence rate have recently been estimated as 133 per 100,000 persons and 83 per 100,0000 person years, respectively [7].

A cohort comparison of PsA patients presenting with early and established disease suggests that delayed diagnosis leads to greater clinical progression [8]. The importance of prompt diagnosis is illustrated by studies that have shown that a several month delay from symptoms onset leads to poor physical function and progressive joint erosion [9]. It should be noted that the lag time from actual disease onset is difficult to pinpoint and accurately describe. The stage at which a patient experiences skin and joint problems does not necessarily imply early PsA, as such findings can be otherwise explained and noncontributory. Two-thirds of PsO patients that report musculoskeletal pain have never seen a rheumatologist and delays in treatment are common [10,11]. Cautious assessment and follow-up by an experienced rheumatologist / dermatologist are warranted; however, there is a real need for reliable biomarkers that could aid in diagnostic and prognostic prediction of psoriatic disease.

## 2. Wide Scope: A Broad Overview on the Pathobiology of Psoriatic Arthritis

Numerous immune cells regulating innate and adaptive responses are increasingly recognized as potential players in the pathogenesis of PsA. Although the interleukin (IL) 23-T helper 17 (Th 17) axis is essential in the development of PsA, there is considerable interface with TNFα and downstream inflammatory signaling. Persistent activation and synergistic interplay between these cytokine pathways could exert effects not only on the skin and joint, but also extend to vasculature and other organs. This may contribute to enhanced progression of atherosclerosis and/or metabolic disease.

It is well established that cardiovascular (CV) and endocrine co-morbidity is a common presentation of psoriatic disease, also present in the spectrum of spondyloarthropathy and systemic inflammatory joint diseases. However, not all inflammation is the same. Different cytokine hubs (e.g., TNFα and the IL-23 – IL-17 axis in psoriatic disease versus IL-6 and TNFα as a central cytokine network in rheumatoid arthritis (RA)) and index condition specific features (e.g., adipocytokines in obesity, inflammaging in elderly, and uremia in chronic kidney disease) may alter the affinity for organ injury, impairment and thus clinical presentation. Studies have shown that gene expression is similar in synovium and skin of PsA patients, but distinct from other rheumatic diseases. Interestingly, the presence of an autoantibody that cross-reacts with an epitope shared by skin and articular antigens has recently been shown to be detectable in 85% of PsA cases (while rare in rheumatoid arthritis (RA) and not present in healthy controls [12]. However, a single, unified molecular pathomechanism for PsA and PsO seems unlikely. Firstly, numerous genes are differentially expressed in paired skin and synovium, even after adjustment for tissue-specific genes [13]. Furthermore, the clinical phenotype and treatment-related response is highly heterogeneous. 

Increasing data from experimental studies suggests that the skin, joint and mucosal surfaces are barrier tissues, which can become the origin point of inflammatory disease in the context of favorable genetic susceptibility and environmental triggers (e.g., trauma, infection and dysbiosis), which lead to immune priming and dysregulation. A theoretical rationale can be drawn from observations that immune barriers (e.g., tight junctions, antimicrobial peptides, immunoglobulin A translocation and immune cell infiltration) in intestinal epithelium are modulated by cytokine signaling, which is thought to prevent pathogen or microbial-byproduct translocation (which in turn would activate immune cells). Leukocyte subpopulations, including neutrophils, macrophages, T effector cell subtypes and other subsets could be the driving populations of inflammation in vascular bed, articular tissue and cutaneous lesions [14,15,16]. 

The gut microbiota is described as the largest endocrine organ, but it is also a modulator of lymphoid tissue. Its function has been tied to a variety of chronic diseases (e.g., obesity, insulin resistance and atherosclerosis) and can be explained as being related to structural and/or functional impairment of the intestinal barrier, alteration of microbiome composition, promotion of pro-inflammatory signaling pathways or generation of harmful metabolites [16,17,18]. Innate immune cells that are sentinels in barrier tissues can be activated in response to injury or pathogens, which can lead to production of factors, such as IL-17, that are responsible for intestinal integrity, secretion of epithelial proteins with antimicrobial activity, and chemokines that recruit neutrophils into endangered tissues [19]. Murine models of psoriasis indicate that the alterations in the microbiome can enable the development of skin disease, while certain commensals could be the source of autoantigens due to molecular mimicry, and thus promote autoreactive T cell activation [18]. 

Dysbiosis is tied to chronic activation of dendritic cells (DCs), which secrete IL-23, activating innate lymphoid cells (ILC) 3s, γδ T cells and natural killer (NK) T cells. Protective effects of IL-17 and IL-22 are imbalanced by proinflammatory TNFα, IL-1 and IL-6 [17]. Dysbiosis characterized by reduction in butyrate-producing bacteria is a feature of inflammatory diseases, including PsA and PsO, which can weaken the physiologic properties of the intestinal barrier and promote antigen stimulation [18]. The process of intestinal inflammation is often linked with increased permeability and translocation of pathogen-associated molecular patterns. It is suspected that extended crosstalk between microbiota and immune subsets occurs. Bacterial DNA can be found in vascular or psoriatic lesions, and distinct microbial presence can be tied to PsO or CV risk [16,17,18]. Certain characteristics of innate immunity (e.g., antimicrobial peptides) are indeed distinct across skin and articular compartments in PsA and PsO [20]. Studies on murine models indicate that environmental triggers, such as dietary intake, may promote IL-17A-producing γδ T cell proliferation and Th17 cytokine expression. IL-23 minicircle delivery was shown to promote lower microbial diversity and dysbiosis. However, following a switch to standard diet, partial regression of these changes was noted [21]. Similarly, the use of antibiotics can suppress gut microbiota and reverse diet induced arterial stiffness and endothelial dysfunction [22]. 

A conceptual framework for the pathobiology of PsA and specific disease phenotypes has recently been reviewed [23,24]. In brief, the articular-dominant disease has been tied to HLA-B*08:01:01, C*07:01:01, which is linked with CD4+ Th1 cell and IL-17+ CD8+ T cell activity, and susceptibility to TNF alpha inhibition. Skin phenotype of PsA was in turn associated with HLA-B*57:01 and HLA-C*06:02, with a driving subset of Th17 cells and potentially greater response to IL-17/23 inhibition. Finally, entheseal (with or without axial involvement) disease can be related to HLA-B*27:05:02, with mixed interactions between CD4+ Th1 cells, IL17+CD8+ T cells and CD4+ Th17 cells [23,24].

Parallels may be drawn between dysfunctional innate and adaptive immune response in PsA and PsO. A combination of genetic susceptibility (e.g., genes for major histocompatibility complex (MHC) class I system, IL-23 and IL-12 beta signaling) and environmental factors (e.g., infection with introduction of specific pathogen associated molecular patterns or trauma with aberrant processes of microinjury and repair) are suspected to incite an aberrant immune cascade [25,26,27,28,29,30]. 

Despite overlapping features, clinical observation shows that skin disease often, but not always, predisposes to arthritic manifestations [28,31,32,33]. Several HLA-B and HLA-C alleles, but also non-HLA genes tied to immune responses (e.g., tied to IL-23 or IL-17 pathways) have been shown to be associated with PsA-specific characteristics. Further, certain similarities to non-psoriatic spondyloarthropathy are apparent in genetic and histological studies (e.g., vascularity, peripheral mononuclear cell presence, lack of intracellular citrullinated protein), thus emphasizing the complexity of PsA etiology [28,34,35,36,37,38]. 

## 3. Genetic Profile May Shape Disease Phenotype

Preference of HLA molecules to bind the positively charged amino acid at position 2 and 3 has been tied to PsA susceptibility, while conversely, B*40:01 and B*44:01 (associated with preference for the negatively charged amino acid position) are considered to have a protective effect. A relationship between HLA-C*06:02 and risk for dominant skin disease with delayed joint involvement is described, while mild skin disease simultaneous with musculoskeletal manifestations could be attributed to B*27:05:02. It appears that the specific HLA genotype can also be tied to some patterns of disease, such as symmetric sacroiliitis, or manifestations of enthesitis and dactylitis, both more common with B*27:05:0. In turn, B*08:01 is linked with asymmetric sacroiliitis [37,39,40]. Examining the genetic profile (e.g., HLA-C*06:02) has also provided evidence that genotype can be tied to treatment response [41,42]. Insertion and deletion in the genome is also of significance for the development of skin disease, and may yield additional explanation for the heterogeneity of manifestations [43]. Cautious interpretation of findings is still necessary as certain confounding factors may be present. For example, recent studies have demonstrated a lack of protective effect of HLA-C*06:02 for PsA [31]. Moreover, studies have examined HLA genetic determinism of treatment response with conflicting results regarding different types of cytokine-inhibiting agents [44]. 

## 4. The IL-23-Th17 Axis Shapes the Molecular Landscape of Skin and Joint Pathology in Psoriatic Arthritis

**Figure 1:**
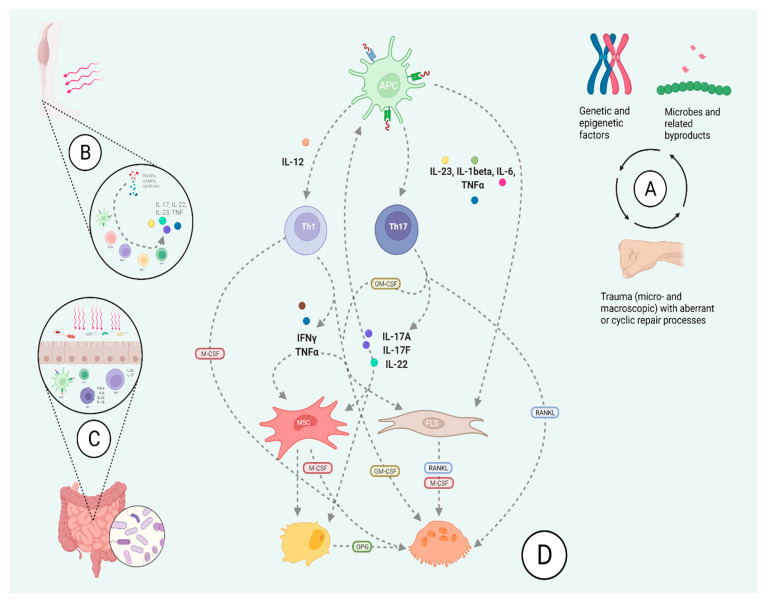
A proposed schematic of psoriatic arthritis pathogenesis with associated points of origin and cellular interactions. **Panel A** shows three main factors that interact with and shape the psoriatic sub-phenotype: (1) genetic susceptibility (e.g. gene alterations involving T-cell activity, IL-23/Th17 signaling or TNF pathways), which could dysregulate an immune response (also mechanisms akin to spondyloarthritis, in which HLA-B27 misfolding can enhance IL-23 production), (2) infection or dysbiosis, which disturbs skin and gut microenvironment and can incite pro-inflammatory responses by contact with pathogen- or damage- associated molecular patterns (PAMPs/DAMPs), (3) biomechanical stress that leads to processes of injury and repair, which, if augmented due to genetic predisposition (e.g., entheseal disease susceptible to low mechanical stress and MHC Class I or IL-23 receptor polymorphisms), could influence resident immune cell populations towards production pro-inflammatory cytokines. **Panel B and C** depict pathological processes in gut and entheseal tissues that could incite, uphold or alter the immune processes leading to PsA (e.g., certain strains can augment Th17 type responses; IL-23 production following endoplasmic reticulum stress can be triggered by bacteria; for a detailed discussion on pathobionts in spondyloarthropathy see [47]). In entheses and/or gut mucosa, triggers such as PAMPs/DAMPs engage a variety of local cells, promote IL-23 production and stimulate immune cell infiltration and production of cytokines. Depending on the tissue, IL-17 and IL-22 may have positive or negative effects on barrier integrity that are weighed with respect to inflammatory TNF-alpha, IL-1 and IL-6 activity. **Panel D** complex interactions between immune cells and microenvironment can lead to bone remodeling (based on [23], more detailed discussion on pathways is provided therein). Antigen-presenting cells release a host of cytokines in response to stimulus (e.g., IL-1beta, IL-6, TNFα, PAMPs/DAMPs) and can also present self-antigens. Th1 and Th17 cells respond and produce several cytokines and growth factors, as depicted, that jointly interact to regulate ongoing processes of osteoblastogenesis/osteoclastogenesis. Bone resorption or new bone formation can alternate depending on the shift between cellular interactions and respective signaling. For example, the effects of cytokines could be stimulating or inhibiting depending on a number of additional factors (e.g., local cytokine milieu or state of cell differentiation).

Immune dysregulation with abnormal cytokine levels and various aberrant immune cell phenotypes is considered to underlie PsA pathogenesis (details in Figure 1) with a central role of the IL-23-Th17 axis [23,24,45]. Immune cell profiling has been shown to differentiate PsA and PsO, which suggests that these conditions have distinct immune cell networks [46]. 

The following references were the basis for the figure and interpretation [17,23,24,45,48].

Th17 cells are T cells characterized by expression of retinoid-related orphan receptor γt (RORγT), regulation by IL-1 and IL-23 signaling and production of IL-17A, IL-17F, IL-21 and IL-22 [15,19,49,50,51]. Studies show IL-17 is a dimer cytokine produced by activated T cells and CD4+CD45RO memory cells. Its production can be stimulated by microbes or cytokines, such as IL-6 or IL-18 [52]. Signal transducer and activator of transcription (STAT) 3 and RORγT signaling are critical in Th17 cell differentiation and production of IL-17A, IL-17F, IL-21 and IL-22. T regulatory/Th17 balance can be suspected to play a role in regulation of immune responses [49,50,51,53]. Several different cell types may produce IL-17 and are often ascribed roles in barrier tissue surveillance. These subsets include NK T cells, lymphoid tissue inducer (LTi) cells, γδ T cells, ILCs and myeloid cells. All may play a role in PsA pathogenesis [19,24].

The local cytokine milieu and interactions with other immune cells (e.g., DCs) lead to a naïve CD4+ T cell shift towards specific populations. IL-17 and IL-22 secreting T cells are present in various tissues from PsA and PsO patients. CD4+ T cells are a likely source of IL-17 and IL-22 in the peripheral blood. Significantly increased frequencies of IL-17+CD4+ T cells and IL-22+CD4+ T cells are observed in arthritic patients, and are associated with IL-17 and IL-22 production [54,55]. Importantly, IL-22 is tied to transition of mesenchymal stem cells into osteoblasts, and is produced by a variety of cell subsets (e.g., γδ T, Th17, Th22) [23]. 

The levels of Th17 cells and IL-17 are strongly linked to disease activity at both early and late stages of PsA (of note, patients with controlled disease have reduced levels). Th17 cells are enhanced in the PsA joint (not only synovial fluid, but also articular tissue and cutaneous lesions in PsA [56]) and characterizing these cells suggests they preferentially migrate to articular areas due to expression of e.g., CCR4 and CCR6 [55]. The Th17 phenotype is important for joint disease in PsA [57,58]. Analyses of the transcriptome from synovium and peripheral blood in PsA support an autoimmune etiology due to upregulation of Th17-related genes, elevated levels of IL-17 producing CD4+ T cells, and higher concentrations of IL-17 and IL-23 in synovium [59]. In the articular compartment, production of IL-17 is reliant on both T cell receptor activation and mesenchymal cell interaction, which contrasts with that of IL-6 or IL-1beta [60]. It has also been shown that synovial tissue is enhanced with polyfunctional Th1, Th17 and exTh17 cells as compared with peripheral blood. These subsets correlate with disease activity. However, the molecular landscape in specific tissues is not clear cut [13,61,62]. Expression of IL-17 is significantly marked in the skin of PsA patients, while the synovium is rather characterized by IL-6, and both sites of pathology seem to be tied to TNFα activity [13].

The synovium in PsA is characterized by angiogenesis, fibroblast activation and inflammatory infiltrates of mononuclear character [57,63]. Synovial gene expression is indeed distinct from skin in matched PsA samples. While TNFα was demonstrated to be homogenously expressed in both tissues, IL23A/IL12B/IL23R was expressed at higher levels in lesional skin (as opposed to non-diseased areas or synovium). Synovial gene expression is apparently highly heterogeneous, which could be a factor explaining clinical differences in treatment response [62]. Moreover, synovial fluid and tissue have distinct cytokine expression in PsA [61,64]. 

Recent studies show that synovial IL-17A+CD8+ T cells share phenotypic and molecular characteristics with Th17 cells in PsA, and have a strong tissue-resident memory T signature and polyfunctional characteristics. They can produce a plethora of pro-inflammatory cytokines (TNF, IL17A, IL-21 and IL-22) [65]. In PsA synovium, CD8+ memory T cells are also more common than in PsO, and their potential significance as a driving subset is emerging [64,66]. Studies have shown that IL-17+ CD8+ T cells are increased in synovial fluid of PsA patients, and may thus exert direct pro-inflammatory and pro-osteoclastogenic effects. This is consistent with observations that frequencies of this subset are tied to clinical, serological and imaging-related characteristics of PsA (including erosive status) [64]. Cutaneous and articular manifestations are postulated to be bridged by interactions of myeloid and lymphoid-derived cells. Synovial fluid of PsA subjects is enriched with toll-like receptor ligands, which could mediate cross-talk of inflammation in skin and joints [67]. Elevated levels of skin derived CD8+CCR10+ T cells have been reported in PsA and are suspected to contribute to PsA development [66]. 

Levels of IL-17 are high in synovial fluid and tissue of patients with inflammatory arthritis [68,69,70]. In comparison with healthy controls, IL-17-producing cells in synovial fluid are significantly enhanced and secretion is correlated with TNFα [71]. Early studies of arthritis models showed that IL-17 can promote inflammation and joint damage both in an IL-1-dependent and independent mechanism [72,73,74,75]. In murine models with IL-17 deficient mice and/or antibody inhibition of IL-17, observations imply an important role for IL-17 in developing synovitis. IL-17 may even mediate the transition from acute macrophage-mediated articular inflammation to T-cell-mediated, chronic arthritis [73,74,76]. Moreover, IL-17 inhibition is effective in reducing joint swelling and cartilage damage in experimental models that are refractory to TNFα inhibition [77]. 

In other organs, such as the lung epithelium, IL-17 can induce chemokines and promote leukocyte infiltration, which points to its pro-inflammatory role in a variety of tissues [51]. IL-17 stimulates epithelial, endothelial and fibroblastic cells towards production of cytokines and chemokines, including IL-6 and IL-8. It can promote expression and synthesis of IL-1beta and TNFα in macrophages, which are crucial cells that infiltrate inflamed articular tissue. IL-17 has also been shown to induce IL-6 and IL-8 production by synovial cells (particularly in initial stages of inflammation), while IL-1beta and IL-17 have synergistic effects of IL-6 production, which itself is another key mediator of joint inflammation [68,78,79,80,81]. IL-17 can also be tied to processes shaping autoimmunity, as for example, it is reported to promote formation and regulation of germinal center structures, by influence of CXC chemokine signaling and Rgs13/16 gene expression in B cells, which could be a mechanism promoting generation of autoreactive antibodies [82]. 

Although reports show that IL-17A levels are not always correlated with disease severity in PsA, high concentrations can be present in synovial fluid, which suggests the importance of a local rather than systemic milieu [54,56]. However, although IL-17A expression is high in synovium, the expression pattern of IL-17A, IL-17F and respective receptors is heterogeneous, which could explain the limited efficacy of IL-17 inhibition in patients belonging to a subgroup with low IL-17 expression [83]. It has recently been demonstrated that neutralizing IL-17A has exceptional clinical effects in the resolution of skin and musculoskeletal (including enthesitis and dactylitis) disease [84,85].

Studies have shown that IL-17A and IL-17F have significant expression in lesional psoriatic skin and in cases of synovitis. The induction of pro-inflammatory mediators by IL-17A and IL-17F is best brought about with synergistic activity of TNF, which emphasizes the importance of interactions between cytokines. Dual neutralization of IL-17A and IL-17F leads to significantly greater inhibition of synoviocytes and dermal fibroblast activation, as opposed to singular inhibition, which is reflected in downregulation of the pro-inflammatory IL-8 and IL-6. Finally, these preclinical observations are confirmed by clinical data that show a rapid and sustained joint and skin response to bimekizumab in active PsA [13,83,86]. 

Research proposes several key interactions between cells of innate and adaptive immunity, as well as regulators of connective tissue and bone turnover. IL-17 production is considerable in synovium from patients with inflammatory arthritis, while it has also been shown to promote gene expression in osteoblasts, which could favor osteoclast differentiation and bone remodeling which are characteristic features of PsA. On a molecular level, it is hypothesized that T cells in the synovium promote cyclooxygenase-2 regulated prostaglandin E2 synthesis in bone resident osteoblast or stromal cells, which in turn induces osteoclast differentiation factor expression and signal transduction for their maturation [68,69]. Patients with PsO have elevated levels of cytokines (e.g., IL-17A and IL-6) and significantly lower bone volume and bony trabeculae, as compared with healthy subjects. Based on data from murine models, IL-17A derived from skin could lead to systemic effects with reduced bone formation. In vitro evidence also shows IL-17A may affect osteoblast and osteocyte activity [87]. Th17 cells have been suspected to alter bone remodeling, potentially via the regulation of osteoclast progenitors and stromal expression of receptor activator for nuclear factor κ B ligand (RANKL). An indirect regulatory effect is suspected to be mediated via IL-17 activity, via stimulation of mesenchymal populations of osteoblasts and fibroblasts, which in turn upregulates RANKL and promotes osteoclastogenesis. IL-17 and other Th-17 related cytokines could also exert inhibitory effects on osteoblast and osteocyte differentiation [87,88]. Notably, IL-17A and IL-17F are potent regulators of osteogenic differentiation and in vitro bone formation based on experimental studies on human periosteal cells [89]. More recent studies have shown that in PsA synovial fluid, IL-17+CD4- T cells are mainly CD8+ T cells. Frequencies of these cells are associated with indices of disease activity and erosive status, though their significance is still unclear [64]. 

Tissue resident memory cells are another cell population of interest, which can persist as a trace element of inflammation when psoriatic skin lesions regress, or could recirculate across tissue via lymphatics. Their potential pathogenicity is underlined by ability to produce IL-17 and IL-22. They could be responsible for initiation or relapse of psoriatic lesions. However, their specific role in PsA is still emerging [66,90].

### 4.1. Moving beyond Singular Cytokine Effects—Synergistic Relationship between TNF Alpha and IL-17

Synergistic effects of IL-17 and TNFα have been shown to affect hepatocyte production of cytokines. However, IL-17 action needs to be antecedent to TNFα activity. Compound activity of TNFα-IL-17 on regulation of keratinocyte genes is also reported [91]. Further, in keratinocytes, IL-23 is induced by synergistic signaling of TNFα, IL-17A and epidermal growth factor [92]. Synergistic induction of pro-inflammatory genes has also been demonstrated in pre-osteoblastic cell lines [93]. Joint IL-17 and TNFα activity is more profound in induction of chemokines such as macrophage inflammatory protein-3 alpha, which can recruit CD4+ memory T cells and facilitate T cell responses [94]. IL-17 and TNFα may thus promote systemic inflammation via IL-6 induction, and IL-6-independent immune cell recruitment (e.g., IL-8-related neutrophil recruitment, chemokine production and DC/Th17 cell recruitment). It has been demonstrated that IL-17 and TNFα lead to expression of TNF type II receptors, which in turn could enhance the response to TNFα and promote production of downstream cytokines such as IL-6 and IL-8 [95]. TNFα, which is considered a master cytokine of innate immunity, also plays an indirect role in the modulation of psoriatic disease. It has been observed that clinical response to TNFα inhibiting agents is reliant on inactivation of myeloid dendritic cell genes and suppression of the Th17 immune pathway [91,96,97,98]. In PsA, TNFα blockade leads to reduction in parameters of systemic inflammation, though IL-17 or IL-23 levels are not reduced, which suggests its role as a component, rather than as a central mediator of cytokine cascades [99]. 

### 4.2. IL-23 Signaling Leads to Psoriatic Skin and Joint Disease

IL-23 is a heterodimer cytokine, which belongs to the IL-12 family and is composed of an IL-12 shared p40 and unique p19 subunit. IL-23 production is ascribed to all antigen-presenting cells, but also neutrophils, epithelial cells and secretory cells. It is an important player in autoimmunity, which promotes Th17 differentiation [17,49,100,101]. IL-23 receptor (IL-23R) activation leads to phosphorylation of protein kinases Jak2 and Tyk2, transcription of STAT3 and RORγ, and finally differentiation of Th17 cells that release IL-17 [17,45]. Studies have examined T cell signal transduction pathway mapping and demonstrated Jak1, STAT3 and STAT1 activation, which appears to mirror an inflammatory process with expansion of T CD4+IL-17+ and T CD4+IL-23R+ Th17 effector cells in synovial fluid of active PsA subjects [102]. Data from murine models also supports the importance of persistent STAT3 activation and development of PsA specific features [88,103]. The clinical relevance is exemplified in successful use of tofacitinib, a Jak inhibitor, for suppression of IL-17 and IFN-gamma production in blood and synovium [45].

IL-17 release by memory T cells is promoted by IL-23, which may lead to induction of chemokines and recruitment of monocyte and neutrophil populations, but also to promotion of co-stimulation and T-cell responses [104]. Both genetic and experimental evidence suggest a role of IL-23, including downstream IL-17 and IL-22 activity, in psoriatic skin disease [105,106,107,108,109,110,111,112]. It is also reported that greater frequencies of T cells expressing IL-23 receptor are present in skin as opposed to peripheral blood of matched PsA samples [54]. Human entheses are also described with a CD14+ myeloid subset, from which IL-23, IL-1beta and TNFα are derived [113]. IL-23 and IL-1beta have been shown to promote the shift from naïve CD4+ T cells towards Th17, in turn leading to production of IL-17A, IL-17F and IL-22, which is viewed as a link between dysregulation of innate and adaptive immunity [49,114,115,116]. The importance of IL-23 in PsA is emphasized by experimental models showing that IL-23 activity alone can lead to the development of phenotypic characteristics like enthesitis and bone remodeling [48]. However, in contrast to IL-22, IL-23 is viewed to have little impact on osteoblastogenesis [23].

It is becoming apparent that IL-23 responding subsets are not limited to Th17 cells, but involve several innate-like T cells (e.g., γδ T cells, MAIT, NKTs) that are found on mucosal, skin and articular surfaces. IL-17 is thus produced by direct innate stimulus or via T cell receptor activation [17]. IL-23 activation of γδ T cells, a major subset of the intraepithelial mucosal and epithelial lymphocyte populations, contributes a refractory profile to Treg suppressive activity, which may enable antigen-specific effector T cell responses [117]. IL-23 can promote expansion, pro-migratory and inflammatory properties of γδT and MAIT cells [17]. Synovial fluid is also enriched with CD8+ MAITs that respond to IL-23, which may in turn lead to joint injury via IL-17 release [118]. Stimulation by IL-23 also leads to increased release of IL-17 and IFN-gamma by Thy1+ innate lymphoid cells (ILCs), which accumulate and promote tissue inflammation. It can be hypothesized that consistent with a diverse range of tissues (e.g., keratinocytes, fibroblasts, epithelium and synovium) expressing IL-22, IL-17, and TNF receptors, Th-17 related cytokines may have deleterious multi-organ effect [50,119]. Notably, ILCs type 3, MAIT cells and γδ T cells have previously been reported as producers of IL-17F and IL-17A, in a manner independent of IL-23 activity [120]. Recent studies also point to the importance of CXCR3+ CD8+ T cells as mediators of joint inflammation. Other reports describe joint enrichment in IL-17+ CD8+ T cells, which is associated with disease activity measures and bone destruction [64,121,122].

Table 1 outlines several clinical questions, for which theoretical justification may be suspected based on the evidence discussed in this paper.

## 5. Inflammation Is Co-Morbidity, but Not all Inflammation Is the Same

A variety of inflammatory mediators and immune cells are ascribed central roles in initiation and resolution of the perpetual cycle of atherosclerosis. IL-1beta, IL-6 and TNF-alpha are considered pro-inflammatory drivers of vascular disease, existing in a perpetual cycle of inflammatory resolution and tissue repair [14,15,16]. Interplay regarding the genetic and immune mechanisms has been reviewed in detail with regard to psoriatic and cardiovascular disease [123]. Aside from lipid disorders, there is a role for immune dysfunction in atherogenesis. Imbalance between Th17 and Treg has been proposed as one of the offending mechanisms. The balance between these cells provides maintenance of self-tolerance and suppresses the development of autoimmunity. The observation of a shift towards elevated Th17 counts and related cytokines (e.g., IL-17, IL-6 and IL-23) can lead to vessel remodeling. In vascular wall lesions, as opposed to healthy vessels, simultaneous secretion of IL-17 and IFN-gamma has been reported, and these cytokine levels are markedly higher than in healthy vessels. Furthermore, synergistic activity of IL-17 and IFN-gamma may be present, with pro-inflammatory stimulation of vascular smooth muscle cells [124]. In endothelial cells, gene expression shows that IL-17 synergism with TNF alpha leads to exceptional enhancement of pro-inflammatory genes for cytokines and chemokines. IL-17 may also lead to endothelial cell changes that promote coagulation and platelet activation [125]. IL-23 is another potential offender with reports indicating mediation of susceptibility to apoptosis in mononuclear cells and promotion of vascular disease progression [126]. It has been hypothesized that mediators of adaptive immunity, such as IFN-gamma, can lead to sensitization of tissue to innate immune activators, which in turn creates a unique inflammatory environment for atherosclerosis [15,127,128,129]. 

Our previous research indicates that in ankylosing spondylitis (AS), another type of inflammatory arthritis, TNFα inhibition leads to some, but not uniform improvement in specific parameters of microvascular function, which is an early, inciting event in the process of vascular disease [130]. Epidemiologic and experimental data suggests that TNFα inhibition could exert positive effects in terms of vascular outcomes. We previously showed that TNFα suppression in inflammatory arthritis may lead to reduced recruitment of inflammatory monocyte subpopulations [131,132]. However, although TNFα inhibition has been shown to reduce endothelial activation, leakage and monocyte adhesion, more recent reports demonstrated reduction in circulating markers of systemic inflammation simultaneously with enhanced signs of atherosclerosis due to a pro-atherogenic lipid profile (increase in vascular markers of inflammation, plaque load in vasculature, decrease in plaque stability) [133,134]. These observations suggest that although circulating parameters of systemic inflammation provide some overview on the burden of systemic inflammation, they do not adequately correspond to disease-specific pathways of inflammation, nor are they reflective of the inflammatory activity in points of origin of inflammation (e.g., immune activation and cytokine levels in synovial fluid can be enhanced in inflamed joints, despite normalization in parameters of systemic inflammation). 

In cohorts with atherosclerotic disease, it has been shown that indirect indices of systemic inflammatory processes (e.g., downstream markers such as C-reactive protein or interleukin-6) are tied to risk of cardiovascular (CV) events. High levels of IL-6 after discharge from hospital are associated with heart failure and reduced left ventricular function [135]. Further reports from clinical trials show that in subjects with prior myocardial infarction, IL-1beta antagonism by canakinumab leads to reduction in CRP and IL-6. Moreover, a potential preventive role for recurrent CV incidents is observed, as compared with placebo [136].

More recent attempts have examined the potential efficacy of low-dose methotrexate, which is a staple disease-modifying antirheumatic drug. The molecular mechanism of methotrexate activity is likely based on suppression of inflammatory signaling via elevation in extracellular adenosine in inflammatory exudate, which leads to occupation of adenosine A2 receptors [137]. However, no reduction in interleukin-1beta, interleukin-6 or CRP was observed, nor was a reduced risk of CV reported. These reports suggest that modulation of the interleukin 1 beta-interleukin 6 pathway is of high interest in prevention of atherosclerosis [138,139]. Indeed, very recent reports from a randomized trial to assess the utility of tocilizumab, an anti-IL-6 agent, have shown that myocardial salvage is significantly improved by cytokine suppression in acute ST elevation myocardial infarction (microvascular obstruction was also lower in the drug arm versus comparator) [140]. Together, these studies emphasize the importance of understanding the mechanistic inflammatory signaling that is characteristic and predominant in a specific condition. This is further illustrated by the high efficacy of methotrexate in rheumatoid arthritis (RA), another systemic inflammatory disease, and the modest benefit of e.g., additional canakinumab treatment.

## 6. Summary 

Environmental triggers, genetic susceptibility and immune dysfunction with a central IL-23 – Th17 axis shape the current understanding of PsA pathobiology. Genetic susceptibility is not only tied to risk of disease development but may also predict the pattern of psoriatic disease progression and organ involvement. Alterations in barrier tissue such as the mucosal surfaces can become sites of immune activation. Dysbiosis can be one of the factors causing predisposition to immune priming and a dysregulated immune response. Inflammatory signature is different across different tissue compartments in PsA. Numerous cell populations are postulated to mediate crosstalk between skin and joints, but the activation of inflammatory cascades can also extend beyond these compartments. On a molecular level, IL-17 can exert pro-inflammatory effects with induction of cytokines and chemokines, but its synergistic interaction with other cytokines, including TNFα, is likely to be a major driver of inflammatory destruction in PsA. IL-17 is elevated in synovial tissue and fluid of inflamed joints. In PsA, the synovium itself is characterized by neovascularization and mononuclear inflammatory infiltrate. Preclinical studies show IL-17 associated inflammation and joint destruction can occur via IL-1-dependent and independent mechanisms. IL-17 inhibition can alleviate arthritis in murine models of arthritis refractory to TNF inhibition. Recent studies point to the importance of IL-17A and IL-17F receptors in synovium as their expression pattern is heterogeneous, which implies certain patients benefit from dual inhibition. IL-23 is a heterodimer cytokine produced by antigen-presenting cells with a key role in autoimmunity and Th17 differentiation. Animal models show that IL-23 is sufficient to induce a PsA-like phenotype and signaling via IL-23R can promote a pro-inflammatory T cell shift. Innate immune responders and the significance of barrier tissue are consistently being explored in PsA pathogenesis, as it becomes apparent that modulating IL-23 and IL-17-related responses extends beyond the conventional cell populations (a detailed summary of experimental studies is provided in Table 2).

## Figures and Tables

**Table 1:** Theoretical explanations to clinical questions of interest based on the studies discussed in this review.

Clinical Questions of Interest	Potential Justification
Are clinical manifestations in PsA consistent with the theorized point of origin?	- Although the entheses are viewed as the origin point of psoriatic disease, clinical manifestations may begin with e.g., dactylitis or axial disease. Typical manifestations are rarely uniform in patient populations, just as arthritis is not always preceded by cutaneous disease. Recent data suggest that PsA that we diagnose clinically is a disease that has been ongoing subclinically for months to years and as such, the characteristic features that we tie to PsA rather represent tissue involvement at a chronic, established phase of disease.
Why is considerable heterogeneity present across spondyloarthritis and even psoriatic arthritis itself?	- Propensity for specific disease phenotype (i.e., the extent and time course of manifestations) seems to be based on genetic susceptibility (e.g., HLA-B and C-alleles; for instance, due to the autoimmune mechanisms associated with misfolding of MHC class complex) and the specifics of the maladaptive immune response (e.g., predominance of cytokine pathways in specific tissue, interactions between affected organs, immune priming). Environmental triggers or genetic changes may alter the antigen presenting cell state, promote inflammatory signaling and result in an autoimmune response to self-peptides. Each individual differs in their repertoire of peptide recognition, and thus heterogeneity in HLA class genes contributes to differential adaptive immune responses, which are further altered by stimulus from the local microenvironment. Interactions between susceptibility alleles may also compound to affect the risk and/or presentation of disease.
What can be responsible for variability in treatment response in PsA?	Clinical problem: Despite several biologic and small molecule drugs being extensively tested in PsA, the response rates remain suboptimal. Even drug changes with respect to cytokine-targets do not always alleviate refractory disease. - It can be suspected that immune pathways in PsA are driven by different combinations of infiltrating and resident cells, with numerous interactions between cells of the microenvironment (e.g., fibroblasts, synoviocytes and mesenchymal cells). Innate responses can trigger the adaptive arm of immunity, which underlies the importance of barrier tissues and the skin and gut microbiome. Synergism between cytokines can augment inflammatory processes. T-cell cytokine functionality should be considered as a potential explanation. Moreover, IL-17-producing cell subsets do not always require IL-23 stimulation, while IL-17A/F receptor expression and cytokine production differ considerably across cells and tissue, which can explain the difficulties with different biologics. An individual’s genotype may also alter interactions between immune subsets and promote signaling through certain pathways. The complex web of immune cell interactions is likely to significantly shift under the effects of targeting a single cytokine, and the change towards other pathways does not simply imply a resolution of disease.
Responses are based on the studies discussed in this review and particularly [1,17,23,24,32,40,48,57,90].	

**Table 2:** Case-by-case summary of selected experimental studies discussed in this review.

Reference	Design	Detailed Summary
Sherlock et al. [48]	Murine model	IL-23 inhibition reduces entheseal inflammation, which is associated with downregulation of cytokines (e.g., IL-6, IL-1beta), chemokines (Cxcl1 and Cxcl2) and factors involved in bone remodeling (Rankl, Ctsk, MMPs). In both axial and peripheral articular surfaces, IL-23R+ resident cells are present in entheses. This population is characterized by “innate-like” responsiveness, which may confer responsiveness to IL-23 in entheses, as seen in the gut.Early on enthesitis is present without synovitis, late in the disease, destructive synovitis and florid enthesitis are present. IL-23 leads to joint inflammation (early changes in entheses and periosteum) and follows a dose-related relationship, though no disease in other organs develops (kidney, liver, gut). IL-23 leads to enthesitis in axial skeleton and sacroiliitis. IL-23 leads to expansion of periosteal osteoblasts, and new entheseal and periosteal cartilage and bone formation. IL-23-driven models do not fully respond to TNF, IL-6 or RANKL inhibition.Th17 is not necessary for IL-23-related inflammatory disease to develop (but rather local IL-23R+ resident cells). IL-23 stimulates IL-17 and IL-22. Inhibition of IL-17 and IL-22 reduces joint swelling, more so in combination. Of note, IL-17 overexpression does not lead to pathology. IL-22 has osteoproliferative effects. Joint swelling with phosphorylation of STAT3 in bone is associated with IL-22. In comparison to IL-23, induction of genes regulating bone formation (Wnt, bone morphogenic proteins, alkaline phosphatase) is more pronounced for IL-22.
Zayyadi et al. [141]	In vitro experiment based on human tissue	Inflammatory stimulus (IFN-gamma and TNF) enhances IL-22 receptor expression in mesenchymal stem cells (MSCs). MSC proliferation and migration are enhanced by concerted activity of IL-22 and inflammatory stimuli. IL-22 upregulates osteogenic markers. When IFN-gamma, TNF and IL-22 act together, chondrogenic and adipogenic transcription factor expression remains largely unaltered, except for reduced elevation of pro-osteogenic RUNX2. IL-22 related osteogenesis is reduced in the presence of inflammatory stimulus (i.e., TNF and IFN-gamma).
Baarsen et al. [83]	In vitro experiment based on human tissue	IL-17A is significantly elevated in synovium of inflammatory arthritis patients, but there is high heterogeneity across individuals. Receptor expression for IL-17A, IL-17F and respective receptors is highly variable in inflammatory synovium. IL-17 producing cells are widespread in synovium, with the majority being CD3+ T cells. CD4+ and CD8+ as well as CD68+ and CD163+ macrophages may be sources of IL-17.
Benham et al. [54]	In vitro experiment based on human tissue	IL-17+ and IL-22+ CD4+ T cells are elevated in peripheral blood mononuclear cells (PBMCs) of both PsA and PsO subjects, as compared with healthy subjects. IL-17 production is enhanced in both PsA and PsO, but IL-22 secretion is greater by PBMCs from PsA subjects (despite similar frequencies of IL-22+ cells). In PsA, increased frequency of CD4+ IL-17+ cells, and reduced CD4+ IL-22+ T cells are observed in synovial fluid. CD4+ IL-17+ T cells in synovial fluid mesenchymal cells were elevated, while CD4+ IL-22+ T cells were reduced (as compared with blood). In synovial tissue, IL-17 is not uniform, while IL-22 expression is absent.
Wade et al. [57]	In vitro experiment based on human tissue	T-cell polyfunctionality with regard to cytokine expression is enhanced in synovial tissue and associated with disease activity, in contrast to monofunctional T cells. PDE4 inhibition leads to suppression of polyfunctional cells.
Menon et al. [64]	In vitro experiment based on human tissue	PsA joints, but not those of RA patients, have enhanced levels of IL-17+ CD4- (CD8+) and IL-17+CD4+T cells. T cell subsets are associated with disease activity and erosive disease status. IL-17+ CD3+ T cells (with increased frequency of CD3+CD4- T cells and CD3+ CD4+ T cells) are elevated in synovial fluid of PsA, as compared to peripheral blood. The majority of IL-17+CD4- T cells are CD8+ or CD161+ T subsets, and a small proportion expressed characteristic markers of MAIT cells or γ/δ cells. Significant differences are observed in cytokine expression of T cells in matched peripheral blood and synovium in PsA. Synovial fluid IL-17+ CD4- T cells, but not CD4+ counterparts, are positively correlated with active synovitis scores. Moreover, IL-22+ CD4- T cells show a similar association. IL-17+CD4- T cells are elevated in synovial fluid from PsA subjects and the proportion of IL-17+ is increased in CD4- and CD4+ T cell populations when erosive disease is present, but not in nonerosive cases.
Baricza et al. [58]	In vitro experiment based on human tissue	Naive CD4+CD45RO− T lymphocytes are shown to be predisposed to shift to Th17 and produce IL-17A and IL-22. Increased RoR γ expression is present in naïve T cells of PsA patients. Cytokine combinations result in specific changes of transcription factors and IL-17A and IL-22 production in PsA. Chemokine receptor patterns suggest naïve T cells are likely to be prematurely engaged in PsA.
Uluckan et al. [87]	Murine model	Increase in Th17 cells is concurrent with reduction in other T helper and regulatory cells (i.e., Th1, Th2 and Treg cells, which may prevent osteoclastogenesis). Osteoclast progenitor cells are likely to accumulate and RANKL may be enhanced due to augmentation of Th17 responses (In the experimental model of R26STAT3Cstopfl/fl CD4Cre mice). Conversely, osteoblasts are characterized by failure to develop. Neutralization of IL-17 or genetic ablation of IL-22 alleviates the psoriasis phenotype. Abrogation of IL-22 and IL-17 (Th17 cytokines) prevents osteopenia.
Xu et al. [142]	In vitro experiment based on human tissue	CD4+ T cells are the major population in PsA synovial fluid and blood (as opposed to CD8+T cells). CD4+ T cells, but not CD8+ T cells are sources of IL-17A in synovial fluid of PsA patients following TCR activation. Anti-17A activity leads to more pronounced inhibition of inflammatory cytokines (e.g., IL-6 and IL-1beta), while TNF-alpha inhibition leads to stronger reduction in MMPs.
Mulder et al. [46]	In vitro experiment based on human tissue	Based on blood-based immune profiling, a reduction in CD4+ and CD8+ memory T-cell subsets, Treg cells and CD196+ and CD197+ monocytes in concert with elevated levels of differentiated CD4+ memory T-cells expressing CCR6 and CCR4 discriminates PsA from PsO. Memory T cells and CCR6+ monocytes are likely to migrate to articular and entheseal tissue, which could explain the differences with PsO peripheral blood. The increase in CD196+ (CCR6) memory T cells is considered to reflect a compensatory proliferation stimulus in response to their efflux to inflamed tissue. CD197+ (CCR7) monocytes are reduced in circulation of PsA subjects, and this subset is strongly associated with disease activity. This may reflect the recruitment of these populations into inflamed tissue, which is also supported by studies that show CCR7 signaling is related to Th-17-driven bone loss.

## References

[1] Kane D., Stafford L., Bresnihan B., FitzGerald O. (2003). A prospective, clinical and radiological study of early psoriatic arthritis: An early synovitis clinic experience. Rheumatology.

[2] Christophers E., Barker J., Griffiths C., Daudén E., Milligan G., Molta C., Sato R., Boggs R. (2010). The risk of psoriatic arthritis remains constant following initial diagnosis of psoriasis among patients seen in European dermatology clinics. J. Eur. Acad. Dermatol. Venereol..

[3] Batko B. (2020). Patient-Centered Care in Psoriatic Arthritis—A Perspective on Inflammation, Disease Activity, and Psychosocial Factors. J. Clin. Med..

[4] Batko B., Kucharz E., Stajszczyk M., Brzosko M., Samborski W., Żuber Z. (2021). Real-World Data from a Multi-Center Study: Insights to Psoriatic Arthritis Care. J. Clin. Med..

[5] Orbai A.M., Reddy S.M., Dennis N., Villacorta R., Peterson S., Mesana L., Chakravarty S.D., Lin I., Karyekar C.S., Wang Y. (2021). Work absenteeism and disability associated with psoriasis and psoriatic arthritis in the USA—A retrospective study of claims data from 2009 to 2020. Clin. Rheumatol..

[6] Wilson F.C., Icen M., Crowson C.S., McEvoy M.T., Gabriel S.E., Kremers H.M. (2009). Incidence and clinical predictors of psoriatic arthritis in patients with psoriasis: A population-based study. Arthritis Rheum..

[7] Scotti L., Franchi M., Marchesoni A., Corrao G. (2018). Prevalence and incidence of psoriatic arthritis: A systematic review and meta-analysis. Semin. Arthritis Rheum..

[8] Gladman D.D., Thavaneswaran A., Chandran V., Cook R.J. (2011). Do patients with psoriatic arthritis who present early fare better than those presenting later in the disease?. Ann. Rheum. Dis..

[9] Haroon M., Gallagher P., FitzGerald O. (2014). Diagnostic delay of more than 6 months contributes to poor radiographic and functional outcome in psoriatic arthritis. Ann. Rheum. Dis..

[10] Felbo S., Terslev L., Sørensen I., Skov L., Zachariae C., Østergaard M. (2021). Musculoskeletal Pain in Patients with Psoriasis and its Influence on Health-related Quality of Life: Results from a Danish Population-based Survey. Acta Derm. Venereol..

[11] Ingrasciotta Y., Isgrò V., Ientile V., Tari M., Trifirò G., Guarneri C. (2021). Are Patients with Psoriasis and Psoriatic Arthritis Undertreated? A Population-Based Study from Southern Italy. J. Clin. Med..

[12] Dolcino M., Lunardi C., Ottria A., Tinazzi E., Patuzzo G., Puccetti A. (2014). Crossreactive Autoantibodies Directed against Cutaneous and Joint Antigens Are Present in Psoriatic Arthritis. PLoS ONE.

[13] Belasco J., Louie J.S., Gulati N., Wei N., Nograles K., Fuentes-Duculan J., Mitsui H., Suárez-Fariñas M., Krueger J.G. (2014). Comparative Genomic Profiling of Synovium Versus Skin Lesions in Psoriatic Arthritis. Arthritis Rheumatol..

[14] Kurilenko N., Fatkhullina A., Mazitova A., Koltsova E. (2021). Act Locally, Act Globally—Microbiota, Barriers, and Cytokines in Atherosclerosis. Cells.

[15] Marchini T., Hansen S., Wolf D. (2021). ApoB-Specific CD4^+^ T Cells in Mouse and Human Atherosclerosis. Cells.

[16] Yeh C.-F., Chen Y.-H., Liu S.-F., Kao H.-L., Wu M.-S., Yang K.-C., Wu W.-K. (2020). Mutual Interplay of Host Immune System and Gut Microbiota in the Immunopathology of Atherosclerosis. Int. J. Mol. Sci..

[17] Łukasik Z., Gracey E., Venken K., Ritchlin C., Elewaut D. (2021). Crossing the boundaries: IL-23 and its role in linking inflammation of the skin, gut and joints. Rheumatology.

[18] Olejniczak-Staruch I., Ciążyńska M., Sobolewska-Sztychny D., Narbutt J., Skibińska M., Lesiak A. (2021). Alterations of the Skin and Gut Microbiome in Psoriasis and Psoriatic Arthritis. Int. J. Mol. Sci..

[19] Cua D.J., Tato C.M. (2010). Innate IL-17-producing cells: The sentinels of the immune system. Nat. Rev. Immunol..

[20] Bierkarre H., Harder J., Cuthbert R., Emery P., Leuschner I., Mrowietz U., Hedderich J., McGonagle D., Gläser R. (2015). Differential expression of antimicrobial peptides in psoriasis and psoriatic arthritis as a novel contributory mechanism for skin and joint disease heterogeneity. Scand. J. Rheumatol..

[21] Shi Z., Wu X., Rocha C.S., Rolston M., Garcia-Melchor E., Huynh M., Nguyen M., Law T., Haas K.N., Yamada D. (2021). Short-Term Western Diet Intake Promotes IL-23–Mediated Skin and Joint Inflammation Accompanied by Changes to the Gut Microbiota in Mice. J. Investig. Dermatol..

[22] Battson M.L., Lee D.M., Jarrell D.K., Hou S., Ecton K.E., Weir T.L., Gentile C.L. (2018). Suppression of gut dysbiosis reverses Western diet-induced vascular dysfunction. Am. J. Physiol. Endocrinol. Metab..

[23] Jadon D.R., Stober C., Pennington S.R., FitzGerald O. (2020). Applying precision medicine to unmet clinical needs in psoriatic disease. Nat. Rev. Rheumatol..

[24] Stober C. (2021). Pathogenesis of psoriatic arthritis. Best Pr. Res. Clin. Rheumatol..

[25] Eder L., Law T., Chandran V., Shanmugarajah S., Shen H., Rosen C.F., Cook R.J., Gladman D.D. (2011). Association between environmental factors and onset of psoriatic arthritis in patients with psoriasis. Arthritis Rheum..

[26] Pattison E., Harrison B.J., Griffiths C.E.M., Silman A.J., Bruce I.N. (2007). Environmental risk factors for the development of psoriatic arthritis: Results from a case-control study. Ann. Rheum. Dis..

[27] Pedersen O.B., Svendsen A.J., Ejstrup L., Skytthe A., Junker P. (2008). On the heritability of psoriatic arthritis. Disease concordance among monozygotic and dizygotic twins. Ann. Rheum. Dis..

[28] Stuart P.E., Nair R.P., Tsoi L.C., Tejasvi T., Das S., Kang H.M., Ellinghaus E., Chandran V., Callis-Duffin K., Ike R. (2015). Genome-wide Association Analysis of Psoriatic Arthritis and Cutaneous Psoriasis Reveals Differences in Their Genetic Architecture. Am. J. Hum. Genet..

[29] Vasey F.B., Deitz C., A Fenske N., Germain B.F., Espinoza L.R. (1982). Possible involvement of group A streptococci in the pathogenesis of psoriatic arthritis. J. Rheumatol..

[30] Filer C., Ho P., Smith R.L., Griffiths C., Young H., Worthington J., Bruce I.N., Barton A. (2008). Investigation of association of the IL12B and IL23R genes with psoriatic arthritis. Arthritis Rheum..

[31] Bowes J., Ashcroft J., Dand N., Jalali-Najafabadi F., Bellou E., Ho P., Marzo-Ortega H., Helliwell P.S., Feletar M., Ryan A. (2017). Cross-phenotype association mapping of the MHC identifies genetic variants that differentiate psoriatic arthritis from psoriasis. Ann. Rheum. Dis..

[32] Gladman D.D., Shuckett R., Russell M.L., Thorne J.C., Schachter R.K. (1987). Psoriatic Arthritis (PSA)—An Analysis of 220 Patients. QJM Int. J. Med..

[33] Pollock R.A., Abji F., Liang K., Chandran V., Pellett F.J., Virtanen C., Gladman D.D. (2015). Gene Expression Differences between Psoriasis Patients with and without Inflammatory Arthritis. J. Investig. Dermatol..

[34] Apel M., Uebe S., Bowes J., Giardina E., Korendowych E., Juneblad K., Pasutto F., Ekici A.B., McManus R., Ho P. (2013). Variants in *RUNX3* Contribute to Susceptibility to Psoriatic Arthritis, Exhibiting Further Common Ground with Ankylosing Spondylitis. Arthritis Rheum..

[35] Eder L., Chandran V., Pellet F., Shanmugarajah S., Rosen C.F., Bull S.B., Gladman D.D. (2011). Human leucocyte antigen risk alleles for psoriatic arthritis among patients with psoriasis. Ann. Rheum. Dis..

[36] Haroon M., Winchester R., Giles J.T., Heffernan E., Fitzgerald O. (2016). Clinical and genetic associations of radiographic sacroiliitis and its different patterns in psoriatic arthritis. Clin. Exp. Rheumatol..

[37] Haroon M., Winchester R., Giles J.T., Heffernan E., FitzGerald O. (2014). Certain class I HLA alleles and haplotypes implicated in susceptibility play a role in determining specific features of the psoriatic arthritis phenotype. Ann. Rheum. Dis..

[38] Kruithof E., Baeten D., De Rycke L., Vandooren B., Foell D., Roth J., Cañete J.D., Boots A.M., Veys E.M., De Keyser F. (2005). Synovial histopathology of psoriatic arthritis, both oligo- and polyarticular, resembles spondyloarthropathy more than it does rheumatoid arthritis. Arthritis Res..

[39] Winchester R., Minevich G., Steshenko V., Kirby B., Kane D., Greenberg D.A., Fitzgerald O. (2011). HLA associations reveal genetic heterogeneity in psoriatic arthritis and in the psoriasis phenotype. Arthritis Rheum..

[40] Winchester R., FitzGerald O. (2020). The many faces of psoriatic arthritis: Their genetic determinism. Rheumatology.

[41] Dand N., Duckworth M., Baudry D., Russell A., Curtis C., Lee S.H., Evans I., Mason K., Alsharqi A., Becher G. (2018). HLA-C*06:02 genotype is a predictive biomarker of biologic treatment response in psoriasis. J. Allergy Clin. Immunol..

[42] Van Vugt L.J., van den Reek J.M.P., Hannink G., Coenen M., De Jong E.M.G.J. (2019). Association of *HLA-C*06:02* Status with Differential Response to Ustekinumab in Patients with Psoriasis: A Systematic Review and Meta-Analysis. JAMA Dermatol..

[43] Zhen Q., Yang Z., Wang W., Li B., Bai M., Wu J., Ge H., Dong Z., Shen J., Tang H. (2019). Genetic Study on Small Insertions and Deletions in Psoriasis Reveals a Role in Complex Human Diseases. J. Investig. Dermatol..

[44] Jiménez C.M., Ramírez C.P., Martín A.S., Maroun S.V., Santiago S.A., Tortosa M.R., Morales A.J. (2021). Influence of Genetic Polymorphisms on Response to Biologics in Moderate-to-Severe Psoriasis. J. Pers. Med..

[45] Carvalho A.L., Hedrich C.M. (2021). The Molecular Pathophysiology of Psoriatic Arthritis—The Complex Interplay between Genetic Predisposition, Epigenetics Factors, and the Microbiome. Front. Mol. Biosci..

[46] Mulder M.L.M., He X., Reek J.M.P.A.V.D., Urbano P.C.M., Kaffa C., Wang X., van Cranenbroek B., van Rijssen E., Hoogen F.H.J.V.D., Joosten I. (2021). Blood-Based Immune Profiling Combined with Machine Learning Discriminates Psoriatic Arthritis from Psoriasis Patients. Int. J. Mol. Sci..

[47] Gill T., Rosenbaum J.T. (2021). Putative Pathobionts in HLA-B27-Associated Spondyloarthropathy. Front. Immunol..

[48] Sherlock J.P., Joyce-Shaikh B., Turner S.P., Chao C.-C., Sathe M., Grein J., Gorman D.M., Bowman E.P., McClanahan T.K., Yearley J.H. (2012). IL-23 induces spondyloarthropathy by acting on ROR-γt^+^ CD3^+^CD4^−^CD8^−^ entheseal resident T cells. Nat. Med..

[49] Harrington L.E., Hatton R.D., Mangan P.R., Turner H., Murphy T.L., Murphy K.M., Weaver C.T. (2005). Interleukin 17–producing CD4+ effector T cells develop via a lineage distinct from the T helper type 1 and 2 lineages. Nat. Immunol..

[50] Ivanov I.I., McKenzie B.S., Zhou L., Tadokoro C.E., Lepelley A., Lafaille J.J., Cua D.J., Littman D.R. (2006). The Orphan Nuclear Receptor RORγt Directs the Differentiation Program of Proinflammatory IL-17+ T Helper Cells. Cell.

[51] Park H., Li Z., Yang X.O., Chang S.H., Nurieva R., Wang Y.-H., Wang Y., Hood L., Zhu Z., Tian Q. (2005). A distinct lineage of CD4 T cells regulates tissue inflammation by producing interleukin 17. Nat. Immunol..

[52] Yao Z., Painter S.L., Fanslow W.C., Ulrich D., MacDuff B.M., Spriggs M.K., Armitage R.J. (1995). Human IL-17: A novel cytokine derived from T cells. J. Immunol..

[53] Harris T.J., Grosso J.F., Yen H.-R., Xin H., Kortylewski M., Albesiano E., Hipkiss E.L., Getnet D., Goldberg M.V., Maris C.H. (2007). Cutting Edge: An in vivo Requirement for STAT3 Signaling in TH17 Development and TH17-Dependent Autoimmunity. J. Immunol..

[54] Benham H., Norris P., Goodall J., Wechalekar M.D., FitzGerald O., Szentpetery A., Smith M., Thomas R., Gaston H. (2013). Th17 and Th22 cells in psoriatic arthritis and psoriasis. Arthritis Res. Ther..

[55] Leipe J., Grunke M., Dechant C., Reindl C., Kerzendorf U., Schulze-Koops H., Skapenko A. (2010). Role of Th17 cells in human autoimmune arthritis. Arthritis Rheum..

[56] Raychaudhuri S.P., Raychaudhuri S.K., Genovese M.C. (2011). IL-17 receptor and its functional significance in psoriatic arthritis. Mol. Cell. Biochem..

[57] Wade S., Canavan M., McGarry T., Low C., Wade S.C., Mullan R.H., Veale D.J., Fearon U. (2019). Association of synovial tissue polyfunctional T-cells with DAPSA in psoriatic arthritis. Ann. Rheum. Dis..

[58] Baricza E., Marton N., Királyhidi P., Kovacs O.T., Székely I.K., Lajkó E., Kőhidai L., Rojkovich B., Érsek B., Buzás E.I. (2018). Distinct in vitro T-Helper 17 Differentiation Capacity of Peripheral Naive T Cells in Rheumatoid and Psoriatic Arthritis. Front. Immunol..

[59] Dolcino M., Ottria A., Barbieri A., Patuzzo G., Tinazzi E., Argentino G., Beri R., Lunardi C., Puccetti A. (2015). Gene Expression Profiling in Peripheral Blood Cells and Synovial Membranes of Patients with Psoriatic Arthritis. PLoS ONE.

[60] Noack M., Ndongo-Thiam N., Miossec P. (2016). Interaction among activated lymphocytes and mesenchymal cells through podoplanin is critical for a high IL-17 secretion. Arthritis Res..

[61] Celis R., Planell N., Fernández-Sueiro J.L., Sanmartí R., Ramírez J., González-Álvaro I., Pablos J.L., Cañete J.D. (2012). Synovial cytokine expression in psoriatic arthritis and associations with lymphoid neogenesis and clinical features. Arthritis Res. Ther..

[62] Nerviani A., Boutet M.-A., Tan W.S.G., Goldmann K., Purkayastha N., Lajtos T.A., Hands R., Lewis M., Kelly S., Pitzalis C. (2020). IL-23 skin and joint profiling in psoriatic arthritis: Novel perspectives in understanding clinical responses to IL-23 inhibitors. Ann. Rheum. Dis..

[63] König A., Krenn V., Gillitzer R., Glöckner J., Jansen E., Gohlke F., Eulert J., Muller-Hermelink H.K. (1997). Inflammatory infiltrate and interleukin-8 expression in the synovium of psoriatic arthritis—An immunohistochemical and mRNA analysis. Rheumatol. Int..

[64] Menon B., Gullick N.J., Walter G.J., Rajasekhar M., Garrood T., Evans H.G., Taams L.S., Kirkham B.W. (2014). Interleukin-17+CD8+ T Cells are Enriched in the Joints of Patients with Psoriatic Arthritis and Correlate with Disease Activity and Joint Damage Progression. Arthritis Rheumatol..

[65] Steel K.J.A., Srenathan U., Ridley M., Durham L.E., Wu S., Ryan S., Hughes C.D., Chan E., Kirkham B.W., Taams L.S. (2019). Polyfunctional, Proinflammatory, Tissue-Resident Memory Phenotype and Function of Synovial Interleukin-17A^+^ CD8^+^ T Cells in Psoriatic Arthritis. Arthritis Rheumatol..

[66] Leijten E.F., van Kempen T.S., Nordkamp M.A.O., Pouw J.N., Kleinrensink N.J., Vincken N.L., Mertens J., Balak D.M.W., Verhagen F.H., Hartgring S.A. (2021). Tissue-Resident Memory CD8+ T Cells from Skin Differentiate Psoriatic Arthritis from Psoriasis. Arthritis Rheumatol..

[67] Van Raemdonck K., Umar S., Palasiewicz K., Romay B., Volkov S., Arami S., Sweiss N., Shahrara S. (2020). TLR7 endogenous ligands remodel glycolytic macrophages and trigger skin-to-joint crosstalk in psoriatic arthritis. Eur. J. Immunol..

[68] Chabaud M., Durand J.M., Buchs N., Page G., Frappart L., Miossec P. (1999). Human interleukin-17: A T cell-derived proinflammatory cytokine produced by the rheumatoid synovium. Arthritis Rheum..

[69] Kotake S., Udagawa N., Takahashi N., Matsuzaki K., Itoh K., Ishiyama S., Saito S., Inoue K., Kamatani N., Gillespie M. (1999). IL-17 in synovial fluids from patients with rheumatoid arthritis is a potent stimulator of osteoclastogenesis. J. Clin. Investig..

[70] Ziolkowska M., Koc A., Luszczykiewicz G., Ksiezopolska-Pietrzak K., Klimczak E., Chwalinska-Sadowska H., Maśliński W. (2000). High Levels of IL-17 in Rheumatoid Arthritis Patients: IL-15 Triggers In Vitro IL-17 Production Via Cyclosporin A-Sensitive Mechanism. J. Immunol..

[71] Infante-Duarte C., Horton H.F., Byrne M.C., Kamradt T. (2000). Microbial Lipopeptides Induce the Production of IL-17 in Th Cells. J. Immunol..

[72] Koenders M.I., Kolls J.K., Oppers-Walgreen B., van den Bersselaar L., Joosten L.A.B., Schurr J.R., Schwarzenberger P., van den Berg W.B., Lubberts E. (2005). Interleukin-17 receptor deficiency results in impaired synovial expression of interleukin-1 and matrix metalloproteinases 3, 9, and 13 and prevents cartilage destruction during chronic reactivated streptococcal cell wall-induced arthritis. Arthritis Rheum..

[73] Lubberts E., Joosten L.A.B., Oppers B., van den Bersselaar L., Coenen-de Roo C.J.J., Kolls J.K., Schwarzenberger P., van de Loo F.A.J., van den Berg W.B. (2001). IL-1-Independent Role of IL-17 in Synovial Inflammation and Joint Destruction during Collagen-Induced Arthritis. J. Immunol..

[74] Lubberts E., Schwarzenberger P., Huang W., Schurr J.R., Peschon J.J., van den Berg W.B., Kolls J.K. (2005). Requirement of IL-17 Receptor Signaling in Radiation-Resistant Cells in the Joint for Full Progression of Destructive Synovitis. J. Immunol..

[75] Nakae S., Saijo S., Horai R., Sudo K., Mori S., Iwakura Y. (2003). IL-17 production from activated T cells is required for the spontaneous development of destructive arthritis in mice deficient in IL-1 receptor antagonist. Proc. Natl. Acad. Sci. USA.

[76] Nakae S., Nambu A., Sudo K., Iwakura Y. (2003). Suppression of Immune Induction of Collagen-Induced Arthritis in IL-17-Deficient Mice. J. Immunol..

[77] Plater-Zyberk C., Joosten L.A.B., A Helsen M.M., I Koenders M., A Baeuerle P., van den Berg W.B. (2008). Combined blockade of granulocyte-macrophage colony stimulating factor and interleukin 17 pathways potently suppresses chronic destructive arthritis in a tumour necrosis factor α-independent mouse model. Ann. Rheum. Dis..

[78] Chabaud M., Fossiez F., Taupin J.L., Miossec P. (1998). Enhancing effect of IL-17 on IL-1-induced IL-6 and leukemia inhibitory factor production by rheumatoid arthritis synoviocytes and its regulation by Th2 cytokines. J. Immunol..

[79] Fossiez F., Djossou O., Chomarat P., Flores-Romo L., Ait-Yahia S., Maat C., Pin J.J., Garrone P., Garcia E., Saeland S. (1996). T cell interleukin-17 induces stromal cells to produce proinflammatory and hematopoietic cytokines. J. Exp. Med..

[80] Jovanovic D.V., A Di Battista J., Martel-Pelletier J., Jolicoeur F.C., He Y., Zhang M., Mineau F. (1998). IL-17 stimulates the production and expression of proinflammatory cytokines, IL-beta and TNF-alpha, by human macrophages. J. Immunol..

[81] Lavocat F., Ndongo-Thiam N., Miossec P. (2017). Interleukin-25 Produced by Synoviocytes Has Anti-inflammatory Effects by Acting as a Receptor Antagonist for Interleukin-17A Function. Front. Immunol..

[82] Hsu H.-C., Yang P., Wang J., Wu Q., Myers R.C., Chen J., Yi J., Guentert T., Tousson A., Stanus A.L. (2007). Interleukin 17–producing T helper cells and interleukin 17 orchestrate autoreactive germinal center development in autoimmune BXD2 mice. Nat. Immunol..

[83] Van Baarsen L.G., Lebre M.C., Van Der Coelen D., Aarrass S., Tang M.W., Ramwadhdoebe T.H., Gerlag D.M., Tak P.P. (2014). Heterogeneous expression pattern of interleukin 17A (IL-17A), IL-17F and their receptors in synovium of rheumatoid arthritis, psoriatic arthritis and osteoarthritis: Possible explanation for nonresponse to anti-IL-17 therapy?. Arthritis Res. Ther..

[84] Gladman D.D., Orbai A.-M., Klitz U., Wei J.C.-C., Gallo G., Birt J., Rathmann S., Shrom D., Marzo-Ortega H. (2019). Ixekizumab and complete resolution of enthesitis and dactylitis: Integrated analysis of two phase 3 randomized trials in psoriatic arthritis. Arthritis Res..

[85] Thaçi D., Blauvelt A., Reich K., Tsai T.-F., Vanaclocha F., Kingo K., Ziv M., Pinter A., Hugot S., You R. (2015). Secukinumab is superior to ustekinumab in clearing skin of subjects with moderate to severe plaque psoriasis: CLEAR, a randomized controlled trial. J. Am. Acad. Dermatol..

[86] Glatt S., Baeten D., Baker T., Griffiths M., Ionescu L., Lawson A.D.G., Maroof A., Oliver R., Popa S., Strimenopoulou F. (2017). Dual IL-17A and IL-17F neutralisation by bimekizumab in psoriatic arthritis: Evidence from preclinical experiments and a randomised placebo-controlled clinical trial that IL-17F contributes to human chronic tissue inflammation. Ann. Rheum. Dis..

[87] Uluçkan Ö., Jimenez M., Karbach S., Jeschke A., Graña O., Keller J., Busse B., Croxford A.L., Finzel S., Koenders M. (2016). Chronic skin inflammation leads to bone loss by IL-17–mediated inhibition of Wnt signaling in osteoblasts. Sci. Transl. Med..

[88] Yang L., Fanok M.H., Mediero-Munoz A., Fogli L.K., Corciulo C., Abdollahi S., Cronstein B.N., Scher J.U., Koralov S.B. (2018). Augmented Th17 Differentiation Leads to Cutaneous and Synovio-Entheseal Inflammation in a Novel Model of Psoriatic Arthritis. Arthritis Rheumatol..

[89] Shah M., Maroof A., Gikas P., Mittal G., Keen R., Baeten D., Shaw S., Roberts S.J. (2020). Dual neutralisation of IL-17F and IL-17A with bimekizumab blocks inflammation-driven osteogenic differentiation of human periosteal cells. RMD Open.

[90] Saczonek A.O., Krajewska-Włodarczyk M., Kasprowicz-Furmańczyk M., Placek W. (2020). Immunological Memory of Psoriatic Lesions. Int. J. Mol. Sci..

[91] Chiricozzi A., Guttman-Yassky E., Suárez-Fariñas M., Nograles K.E., Tian S., Cardinale I., Chimenti S., Krueger J.G. (2011). Integrative Responses to IL-17 and TNF-α in Human Keratinocytes Account for Key Inflammatory Pathogenic Circuits in Psoriasis. J. Investig. Dermatol..

[92] Ehst B., Wang Z., Leitenberger J., McClanahan D., De La Torre R., Sawka E., Ortega-Loayza A.G., Strunck J., Greiling T., Simpson E. (2020). Synergistic induction of IL-23 by TNFα, IL-17A, and EGF in keratinocytes. Cytokine.

[93] Shen F., Ruddy M.J., Plamondon P., Gaffen S.L. (2004). Cytokines link osteoblasts and inflammation: Microarray analysis of interleukin-17- and TNF-α-induced genes in bone cells. J. Leukoc. Biol..

[94] Chabaud M., Page G., Miossec P. (2001). Enhancing Effect of IL-1, IL-17, and TNF-α on Macrophage Inflammatory Protein-3α Production in Rheumatoid Arthritis: Regulation by Soluble Receptors and Th2 Cytokines. J. Immunol..

[95] Beringer A., Thiam N., Molle J., Bartosch B., Miossec P. (2018). Synergistic effect of interleukin-17 and tumour necrosis factor-α on inflammatory response in hepatocytes through interleukin-6-dependent and independent pathways. Clin. Exp. Immunol..

[96] Zaba L.C., Suárez-Fariñas M., Fuentes-Duculan J., Nograles K.E., Guttman-Yassky E., Cardinale I., Lowes M.A., Krueger J.G. (2009). Effective treatment of psoriasis with etanercept is linked to suppression of IL-17 signaling, not immediate response TNF genes. J. Allergy Clin. Immunol..

[97] Johnston A., Fritz Y., Dawes S.M., Diaconu D., Al-Attar P.M., Guzman A.M., Chen C.S., Fu W., Gudjonsson J.E., McCormick T.S. (2013). Keratinocyte Overexpression of IL-17C Promotes Psoriasiform Skin Inflammation. J. Immunol..

[98] Najm A., McInnes I.B. (2021). IL-23 orchestrating immune cell activation in arthritis. Rheumatology.

[99] Melis L., Vandooren B., Kruithof E., Jacques P., De Vos M., Mielants H., Verbruggen G., De Keyser F., Elewaut D. (2009). Systemic levels of IL-23 are strongly associated with disease activity in rheumatoid arthritis but not spondyloarthritis. Ann. Rheum. Dis..

[100] Becher B., Durell B.G., Noelle R.J. (2002). Experimental autoimmune encephalitis and inflammation in the absence of interleukin-12. J. Clin. Investig..

[101] Oppmann B., Lesley R., Blom B., Timans J.C., Xu Y., Hunte B., Vega F., Yu N., Wang J., Singh K. (2000). Novel p19 Protein Engages IL-12p40 to Form a Cytokine, IL-23, with Biological Activities Similar as Well as Distinct from IL-12. Immunity.

[102] Fiocco U., Accordi B., Martini V., Oliviero F., Facco M., Cabrelle A., Piva L., Molena B., Caso F., Costa L. (2014). JAK/STAT/PKCδ molecular pathways in synovial fluid T lymphocytes reflect the in vivo T helper-17 expansion in psoriatic arthritis. Immunol. Res..

[103] Fogli L.K., Sundrud M.S., Goel S., Bajwa S., Jensen K., Derudder E., Sun A., Coffre M., Uyttenhove C., Van Snick J. (2013). T Cell–Derived IL-17 Mediates Epithelial Changes in the Airway and Drives Pulmonary Neutrophilia. J. Immunol..

[104] Aggarwal S., Ghilardi N., Xie M.-H., de Sauvage F.J., Gurney A.L. (2003). Interleukin-23 Promotes a Distinct CD4 T Cell Activation State Characterized by the Production of Interleukin-17. J. Biol. Chem..

[105] Capon F., Di Meglio P., Szaub J., Prescott N., Dunster C., Baumber L., Timms K., Gutin A., Abkevic V., Burden A.D. (2007). Sequence variants in the genes for the interleukin-23 receptor (IL23R) and its ligand (IL12B) confer protection against psoriasis. Hum. Genet..

[106] Cargill M., Schrodi S., Chang M., Garcia V.E., Brandon R., Callis K.P., Matsunami N., Ardlie K.G., Civello D., Catanese J.J. (2007). A Large-Scale Genetic Association Study Confirms IL12B and Leads to the Identification of IL23R as Psoriasis-Risk Genes. Am. J. Hum. Genet..

[107] Chan J.R., Blumenschein W., Murphy E., Diveu C., Wiekowski M., Abbondanzo S., Lucian L., Geissler R., Brodie S., Kimball A. (2006). IL-23 stimulates epidermal hyperplasia via TNF and IL-20R2–dependent mechanisms with implications for psoriasis pathogenesis. J. Exp. Med..

[108] Lee E., Trepicchio W.L., Oestreicher J.L., Pittman D., Wang F., Chamian F., Dhodapkar M., Krueger J.G. (2004). Increased Expression of Interleukin 23 p19 and p40 in Lesional Skin of Patients with Psoriasis Vulgaris. J. Exp. Med..

[109] Nair R.P., Duffin K.C., Helms C., Ding J., Stuart P.E., Goldgar D., Gudjonsson J.E., Li Y., Tejasvi T., Feng B.-J. (2009). Genome-wide scan reveals association of psoriasis with IL-23 and NF-κB pathways. Nat. Genet..

[110] Nikamo P., Lysell J., Ståhle M. (2015). Association with Genetic Variants in the IL-23 and NF-κB Pathways Discriminates between Mild and Severe Psoriasis Skin Disease. J. Investig. Dermatol..

[111] Rizzo H.L., Kagami S., Phillips K.G., Kurtz S.E., Jacques S.L., Blauvelt A. (2010). IL-23–Mediated Psoriasis-Like Epidermal Hyperplasia Is Dependent on IL-17A. J. Immunol..

[112] Tonel G., Conrad C., Laggner U., Di Meglio P., Grys K., McClanahan T.K., Blumenschein W.M., Qin J.-Z., Xin H., Oldham E. (2010). Cutting Edge: A Critical Functional Role for IL-23 in Psoriasis. J. Immunol..

[113] Bridgewood C., Watad A., Russell T., Palmer T.M., Marzo-Ortega H., Khan A., A Millner P., Dunsmuir R., Rao A., Loughenbury P. (2019). Identification of myeloid cells in the human enthesis as the main source of local IL-23 production. Ann. Rheum. Dis..

[114] Ghoreschi K., Laurence A., Yang X.-P., Tato C.M., McGeachy M.J., Konkel J.E., Ramos H.L., Wei L., Davidson T.S., Bouladoux N. (2010). Generation of pathogenic TH17 cells in the absence of TGF-β signalling. Nature.

[115] Stockinger B., Veldhoen M., Martin B. (2007). Th17 T cells: Linking innate and adaptive immunity. Semin. Immunol..

[116] Wilson N.J., Boniface K., Chan J.R., McKenzie B.S., Blumenschein W.M., Mattson J.D., Basham B., Smith K.J., Chen T., Morel F. (2007). Development, cytokine profile and function of human interleukin 17–producing helper T cells. Nat. Immunol..

[117] Petermann F., Rothhammer V., Claussen M.C., Haas J.D., Blanco L.R., Heink S., Prinz I., Hemmer B., Kuchroo V.K., Oukka M. (2010). γδ T Cells Enhance Autoimmunity by Restraining Regulatory T Cell Responses via an Interleukin-23-Dependent Mechanism. Immunity.

[118] Raychaudhuri S.K., Abria C., Mitra A., Raychaudhuri S.P. (2019). Functional significance of MAIT cells in psoriatic arthritis. Cytokine.

[119] Buonocore S., Ahern P.P., Uhlig H.H., Ivanov I.I., Littman D.R., Maloy K.J., Powrie F. (2010). Innate lymphoid cells drive interleukin-23-dependent innate intestinal pathology. Nature.

[120] Cole S., Murray J., Simpson C., Okoye R., Tyson K., Griffiths M., Baeten D., Shaw S., Maroof A. (2020). Interleukin (IL)-12 and IL-18 Synergize to Promote MAIT Cell IL-17A and IL-17F Production Independently of IL-23 Signaling. Front. Immunol..

[121] Diani M., Casciano F., Marongiu L., Longhi M., Altomare A., Pigatto P.D., Secchiero P., Gambari R., Banfi G., Manfredi A.A. (2019). Increased frequency of activated CD8+ T cell effectors in patients with psoriatic arthritis. Sci. Rep..

[122] Penkava F., Velasco-Herrera M.D.C., Young M.D., Yager N., Nwosu L.N., Pratt A.G., Lara A.L., Guzzo C., Maroof A., Mamanova L. (2020). Single-cell sequencing reveals clonal expansions of pro-inflammatory synovial CD8 T cells expressing tissue-homing receptors in psoriatic arthritis. Nat. Commun..

[123] Wierzbowska-Drabik K., Lesiak A., Skibińska M., Niedźwiedź M., Kasprzak J.D., Narbutt J. (2021). Psoriasis and Atherosclerosis—Skin, Joints, and Cardiovascular Story of Two Plaques in Relation to the Treatment with Biologics. Int. J. Mol. Sci..

[124] Eid R.E., Rao D.A., Zhou J., Lo S.-F.L., Ranjbaran H., Gallo A., Sokol S.I., Pfau S., Pober J.S., Tellides G. (2009). Interleukin-17 and Interferon-γ Are Produced Concomitantly by Human Coronary Artery–Infiltrating T Cells and Act Synergistically on Vascular Smooth Muscle Cells. Circulation.

[125] Hot A., Lenief V., Miossec P. (2012). Combination of IL-17 and TNFα induces a pro-inflammatory, pro-coagulant and pro-thrombotic phenotype in human endothelial cells. Ann. Rheum. Dis..

[126] Subramanian M., Thorp E., Tabas I. (2015). Identification of a Non-Growth Factor Role for GM-CSF in Advanced Atherosclerosis: Promotion of Macrophage Apoptosis and Plaque Necrosis through IL-23 Signaling. Circ. Res..

[127] Ahmad U., Ali R., Lebastchi A.H., Qin L., Lo S.-F.L., Yakimov A.O., Khan S.F., Choy J., Geirsson A., Pober J.S. (2010). IFN-γ Primes Intact Human Coronary Arteries and Cultured Coronary Smooth Muscle Cells to Double-Stranded RNA- and Self-RNA–Induced Inflammatory Responses by Upregulating TLR3 and Melanoma Differentiation-Associated Gene 5. J. Immunol..

[128] Cheng X., Yu X., Ding Y.-J., Fu Q.-Q., Xie J.-J., Tang T.-T., Yao R., Chen Y., Liao Y.-H. (2008). The Th17/Treg imbalance in patients with acute coronary syndrome. Clin. Immunol..

[129] Xie J.-J., Wang J., Tang T.-T., Chen J., Gao X.-L., Yuan J., Zhou Z.-H., Liao M.-Y., Yao R., Yu X. (2010). The Th17/Treg functional imbalance during atherogenesis in ApoE^−/−^ mice. Cytokine.

[130] Batko B., Maga P., Urbanski K., Ryszawa-Mrozek N., Schramm-Luc A., Koziej M., Mikolajczyk T., McGinnigle E., Czesnikiewicz-Guzik M., Ceranowicz P. (2018). Microvascular dysfunction in ankylosing spondylitis is associated with disease activity and is improved by anti-TNF treatment. Sci. Rep..

[131] Ahmed A., Hollan I., Curran S.A., Kitson S.M., Riggio M.P., Mikkelsen K., Almdahl S.M., Aukrust P., McInnes I.B., Goodyear C.S. (2016). Brief Report: Proatherogenic Cytokine Microenvironment in the Aortic Adventitia of Patients with Rheumatoid Arthritis. Arthritis Rheumatol..

[132] Batko B., Schramm-Luc A., Skiba D.S., Mikolajczyk T.P., Siedlinski M. (2019). TNF-α Inhibitors Decrease Classical CD14hiCD16− Monocyte Subsets in Highly Active, Conventional Treatment Refractory Rheumatoid Arthritis and Ankylosing Spondylitis. Int. J. Mol. Sci..

[133] Oberoi R., Schuett J., Schuett H., Koch A.-K., Luchtefeld M., Grote K., Schieffer B. (2016). Targeting Tumor Necrosis Factor-α with Adalimumab: Effects on Endothelial Activation and Monocyte Adhesion. PLoS ONE.

[134] Oberoi R., Vlacil A.-K., Schuett J., Schösser F., Schuett H., Tietge U.J., Schieffer B., Grote K. (2018). Anti-tumor necrosis factor-α therapy increases plaque burden in a mouse model of experimental atherosclerosis. Atherosclerosis.

[135] Gabriel A.S., Martinsson A., Wretlind B., Ahnve S. (2004). IL-6 levels in acute and post myocardial infarction: Their relation to CRP levels, infarction size, left ventricular systolic function, and heart failure. Eur. J. Intern. Med..

[136] Ridker P.M., Everett B.M., Thuren T., MacFadyen J.G., Chang W.H., Ballantyne C., Fonseca F., Nicolau J., Koenig W., Anker S.D. (2017). Antiinflammatory Therapy with Canakinumab for Atherosclerotic Disease. N. Engl. J. Med..

[137] Cronstein B., Naime D., Ostad E. (1993). The antiinflammatory mechanism of methotrexate. Increased adenosine release at inflamed sites diminishes leukocyte accumulation in an in vivo model of inflammation. J. Clin. Investig..

[138] Alten R., Gómez-Reino J., Durez P., Beaulieu A., Sebba A., Krammer G., Preiss R., Arulmani U., Widmer A., Gitton X. (2011). Efficacy and safety of the human anti-IL-1beta monoclonal antibody canakinumab in rheumatoid arthritis: Results of a 12-week, phase II, dose-finding study. BMC Musculoskelet. Disord..

[139] Ridker P.M., Everett B.M., Pradhan A., MacFadyen J.G., Solomon D.H., Zaharris E., Mam V., Hasan A., Rosenberg Y., Iturriaga E. (2019). Low-Dose Methotrexate for the Prevention of Atherosclerotic Events. N. Engl. J. Med..

[140] Broch K., Anstensrud A.K., Woxholt S., Sharma K., Tøllefsen I.M., Bendz B., Aakhus S., Ueland T., Amundsen B.H., Damås J.K. (2021). Randomized Trial of Interleukin-6 Receptor Inhibition in Patients With Acute ST-Segment Elevation Myocardial Infarction. J. Am. Coll. Cardiol..

[141] El-Zayadi A.A., Jones E.A., Churchman S.M., Baboolal T., Cuthbert R.J., El-Jawhari J., Badawy A.M., Alase A.A., El-Sherbiny Y., McGonagle D. (2016). Interleukin-22 drives the proliferation, migration and osteogenic differentiation of mesenchymal stem cells: A novel cytokine that could contribute to new bone formation in spondyloarthropathies. Rheumatology.

[142] Xu X., Davelaar N., Mus A., Asmawidjaja P.S., Hazes J.M.W., Baeten D.L.P., Vis M., Bisoendial R.J., Prens E.P., Lubberts E. (2020). Interleukin-17A Is Produced by CD4+ but Not CD8+ T Cells in Synovial Fluid Following T Cell Receptor Activation and Regulates Different Inflammatory Mediators Compared to Tumor Necrosis Factor in a Model of Psoriatic Arthritis Synovitis. Arthritis Rheumatol..

